# Effectiveness of a personalized e-coaching intervention on nutritional behavior and eating self-regulation among adults with overweight or obesity: The PRALIMAP-CINeCO randomized trial

**DOI:** 10.1016/j.invent.2026.101019

**Published:** 2026-10-30

**Authors:** Florian Manneville, Johanne Langlois, Philip Böhme, Mélanie Béguinet, Willy Ngueyon Sime, Elisabeth Spitz, Serge Briançon, Edith Lecomte, Abdou Y. Omorou

**Affiliations:** a Université de Lorraine, Inserm, INSPIIRE, F-54000, Nancy, France; b National Conservatory of Arts and Crafts (CNAM), Nancy, France; c Department of Diabetology, Metabolic Diseases and Nutrition, Nancy University Hospital, Nancy, France; d CHRU-Nancy, INSERM, Université de Lorraine, CIC, Epidémiologie Clinique, F-54000, Nancy, France

**Keywords:** Health e-coaching, Professional guidance, Obesity, Young adult, Intervention, Nutritional behaviors

## Abstract

**Background:**

Interventions targeting eating behavior, physical activity, sedentary behavior, and eating self-regulation demonstrate promise for improving weight loss and preventing weight gain. Multifaceted health e-coaching could enhance the effectiveness of such interventions; however, there is limited empirical evidence of its effectiveness.

**Objective:**

To evaluate the effectiveness of a health e-coaching (HeC) intervention compared to standard care (SC) on changes in eating behavior, physical activity, sedentary behavior, and eating self-regulation among young adults with overweight or obesity.

**Methods:**

The primary outcome of this randomized trial included improvement in at least two of three components: eating behavior; physical activity and sedentary behavior; and eating self-regulation strategies. The HeC intervention consisted of (1) online asynchronous individual activities and (2) teleconsultations with health professionals. Comparisons between groups were conducted on an intention-to-treat basis by using logistic regression models.

**Results:**

A total of 100 young adults were followed up at the end of the intervention (49 in the HeC group and 51 in the SC group). At baseline, the mean age was 24.5 ± 5.1 years, and 69% of the participants were women. Intention-to-treat analysis revealed that participants in the HeC group were 3.2 times (95% confidence interval [1.2; 8.2]) more likely to achieve the primary outcome than participants in the SC group.

**Conclusions:**

The e-coaching intervention was effective at improving nutritional behaviors among young adults who were with overweight or obesity. This study provides valuable insights for the future implementation of scalable e-coaching programs in real-world settings.

Trial Registration: NCT05399407.

## Highlights

• This study evaluates a multifaceted health e-coaching intervention.
• The intervention improved eating and sedentary behavior outcomes.
• Effects were smaller for physical activity and eating self-regulation.
• Findings support scalable e-coaching programs in real-world settings.

## 1 Introduction

Obesity is recognized as a major global public health concern, with 20.3% of individuals aged 15–24 years and 45.1% of the total adult population aged 25 years and older being with overweight or obesity (Kerr et al., 2025; Ng et al., 2025). In France, the analysis is similar, with 48.8% of adults having been reported to be with overweight or obesity in 2024 (Ligue nationale contre l'obésité, 2024). Early-onset overweight or obesity is associated with elevated risks of type 2 diabetes, hypertension, cardiovascular disease, and various types of cancers that persist across the lifespan (Zhou et al., 2024). Therefore, young adulthood (the period following adolescence that is characterized by transitions in autonomy, social roles, and behaviors) appears to be a critical window for overweight and obesity prevention (Bonnie et al., 2015).

Key behavioral determinants of overweight and obesity include eating behavior, physical activity, and sedentary behavior. High intake of energy-dense foods and unhealthy eating habits were identified as significant risk factors for obesity among young adults in a scoping review (Lee et al., 2023). In a meta-analysis, Jayedi et al. demonstrated that every 30 min of aerobic exercise per week was associated with a reduction in body weight of 0.52 kg, a reduction in waist circumference of 0.56 cm, and a reduction in body fat percentage of 0.37% (Jayedi et al., 2024). In another meta-analysis, the authors reported that sedentary behavior increased the risk of obesity in adults by 45% (Silveira et al., 2022). Recent findings indicate that the self-regulation of eating, such as through the management of existing temptations, has emerged as an important determinant of healthy eating behaviors (Lopez et al., 2026). Therefore, interventions that explicitly target these health-related behaviors and self-regulatory skills demonstrate promise in improving weight loss and preventing weight gain.

Recent advances in digital health technologies and e-coaching offer new opportunities for scalable, personalized health interventions (Yeung et al., 2023). Personalized e-coaching interventions include several key components: content tailored to individual characteristics and/or customized feedback on health-related behaviors (Ordovas et al., 2018; Alder, 2007); one or more behavior change techniques (Michie et al., 2011); and delivery through digital channels such as websites, mobile phone applications, SMS, or email (Lau et al., 2020). In this way, personalized health e-coaching could integrate behavior change techniques in an interactive fashion, thereby providing feedback, reminders, monitoring, and motivational support; moreover, it could enhance changes in health-related behaviors and self-regulatory skills in a remote manner.

Multifaceted personalized health e-coaching that addresses several complementary obesity-related domains (i.e., physical activity, eating behavior, and psychology), offers a variety of asynchronous digital activity formats, and includes individualized support from health professionals such as dieticians, professionals specialized in adapted physical activity, and psychologists could improve the effectiveness, adherence and applicability of obesity treatment and prevention interventions. While the literature suggests that personalized health e-coaching interventions can support weight loss and weight loss maintenance in adults with overweight or obesity (Kupila et al., 2023), systematic reviews have highlighted the potential value of multifaceted personalized health e-coaching interventions from a multidisciplinary team, including psychologists, nutritionists, exercise therapists, and biomedical scientists (Lau et al., 2020; Spring et al., 2021), and the lack of studies for such intervention targeting young adults (Willmott et al., 2019). Therefore, the primary aim of the present study was to evaluate the effectiveness of a multifaceted personalized health e-coaching (HeC) intervention compared to standard care (SC) on changes in eating behavior, physical activity, sedentary behavior, and eating self-regulation among young adults with overweight or obesity. Secondary objectives examined the effectiveness of HeC compared with SC on changes in body mass index (BMI), eating disorders, anxiety, depression, and quality of life (QoL).

## 2 Methods

### 2.1 Design, setting and participants of the PRALIMAP-CINeCO trial

PRALIMAP-CINeCO was a superiority randomized controlled trial conducted in France from 2023 to 2024 among young adults. This trial is an extension of the follow-up of adolescents who participated in the PRALIMAP-INÈS trial (Legrand et al., 2017). PRALIMAP-INÈS (conducted between 2012 and 2015 in French schools) aimed to demonstrate that tailored support for adolescents with overweight or obesity from less advantaged socioeconomic statuses has an equivalent effect to standard support for adolescents from advantaged socioeconomic statuses in the short term on weight status. Ten years later, the 1419 participants who had previously participated in the trial were recontacted by using the addresses and telephone numbers recorded in the PRALIMAP-INÈS database. Prior to telephone contact, an information letter was mailed to their last known address to introduce the PRALIMAP-CINeCO study and inform them of the upcoming call. A trained clinical research technician then contacted each participant via telephone to explain the study and invite them to attend a medical visit. Those individuals who agreed to participate were asked to complete an online questionnaire and to attend a medical visit with a physician, a clinical research nurse, or a primary care nurse who was specifically trained in clinical research. The questionnaire and medical visit aimed to describe the future of young adults since their participation in the PRALIMAP-INÈS trial. During the medical visit, anthropometric measurements were obtained, and the participants were formally invited to join PRALIMAP-CINeCO. Inclusion (T0) was confirmed after a study physician reviewed the inclusion criteria (see the ‘Study population’ section). The included young adults were then randomized into one of the two intervention groups for 6 to 12 months until the end of the PRALIMAP-CINeCO intervention (T1). Young adults were informed of their group assignment at the end of the medical visit.

Despite multiple attempts to contact the 1419 former PRALIMAP-INÈS participants (via phone calls, text messages, letters, and emails), only 82 agreed to complete the questionnaire and attend medical visits. Of these participants, 37 were eligible to participate in the PRALIMAP-CINeCO trial. To compensate for this low recruitment rate, enrollment was extended to students from the University of Lorraine, as they are young adults. Participation in the study was entirely voluntary. Communication about the study was performed within the university (via student health services, information spots on faculty display screens, flyers, articles in the university press, and information stands in the university library) to inform students about the study. Interested students could scan a QR code on the communication materials to provide their contact details and to receive communication from the clinical research technicians. The subsequent procedure was identical to that applied to the former PRALIMAP-INÈS participants.

### 2.2 Study population

The eligibility of participants contacted was assessed based on the following inclusion criteria: (1) had received complete study information and did not object to participation; (2) completed the preliminary questionnaire before or during the baseline visit (T0); (3) accepted noninvasive anthropometric measurements at T0; (4) exhibited a BMI > 25 kg/m^2^ (Cole et al., 2000) without receiving ongoing medical management for this condition; (5) agreed to participate in the follow-up and to be randomized to either the HeC or the SC group; (6) were affiliated with a social security scheme; and (7) resided in France. For volunteer students, additional criteria included (8) being enrolled at the University of Lorraine and (9) being 18 years of age or older. There were no exclusion criteria.

### 2.3 Interventions

After the inclusion visit (T0), each participant received a summary letter containing the data from their visit (obtained via the self-administered questionnaire and clinical measurements, including BMI) and their group assignment.

#### 2.3.1 Health e-coaching

The PRALIMAP-CINeCO HeC intervention is an innovative and customizable intervention for young adults who are with overweight or obesity and is based on patient therapeutic education principles. It offers two complementary forms of support: (1) asynchronous individual activities hosted on an online learning platform (Fig. 1) and (2) regular teleconsultations with dietitians, psychologists, and adapted physical activity specialists. Each participant completed a baseline questionnaire assessing knowledge and skills, after which they were invited to participate in an initial shared educational assessment. Participants' needs were identified through the completed questionnaires and a shared educational assessment conducted by a randomly assigned point-of-contact health professional (dietitian, psychologist, or physical activity specialist). Needs were based on the levels assessed across several dimensions, including health-related behaviors, knowledge and skills, anxiety and depressive states, emotional regulation, motivations, and social barriers (e.g., lack of time, limited social support, and financial constraints). The identified needs were entered into an Excel spreadsheet designed to support the development of a personalized program. Based on these needs, the spreadsheet generated recommendations for activities (classified as either highly recommended or suggested) and topics to be addressed during teleconsultations with health professionals. The proposed program was then discussed and refined collaboratively by the point-of-contact health professional and the young adult, taking into account the participant's preferences and individual circumstances (e.g., availability). Once agreed upon, the finalized program was emailed to the young adult, including a summary of the planned activities and details of upcoming teleconsultations. A follow-up shared educational assessment was conducted online during the intervention to review progress and adjust the program (if needed). The platform provides interactive tools such as a user guide, discussion forum, personal health journal, and direct contact with professionals. More than 80 engaging activities are distributed across 12 modules, encompassing general health, physical activity, nutrition, and psychological dimensions. Activities include web series, tutorials, serious games, quizzes, and testimonials, along with self-assessment, reflection, goal-setting, planning, and personal resource identification exercises. To foster engagement, participants can earn up to 28 virtual badges reflecting their progress. User-friendly navigation is ensured through color codes, icons, event calendars, and real-time progress tracking.

**Fig. 1:**
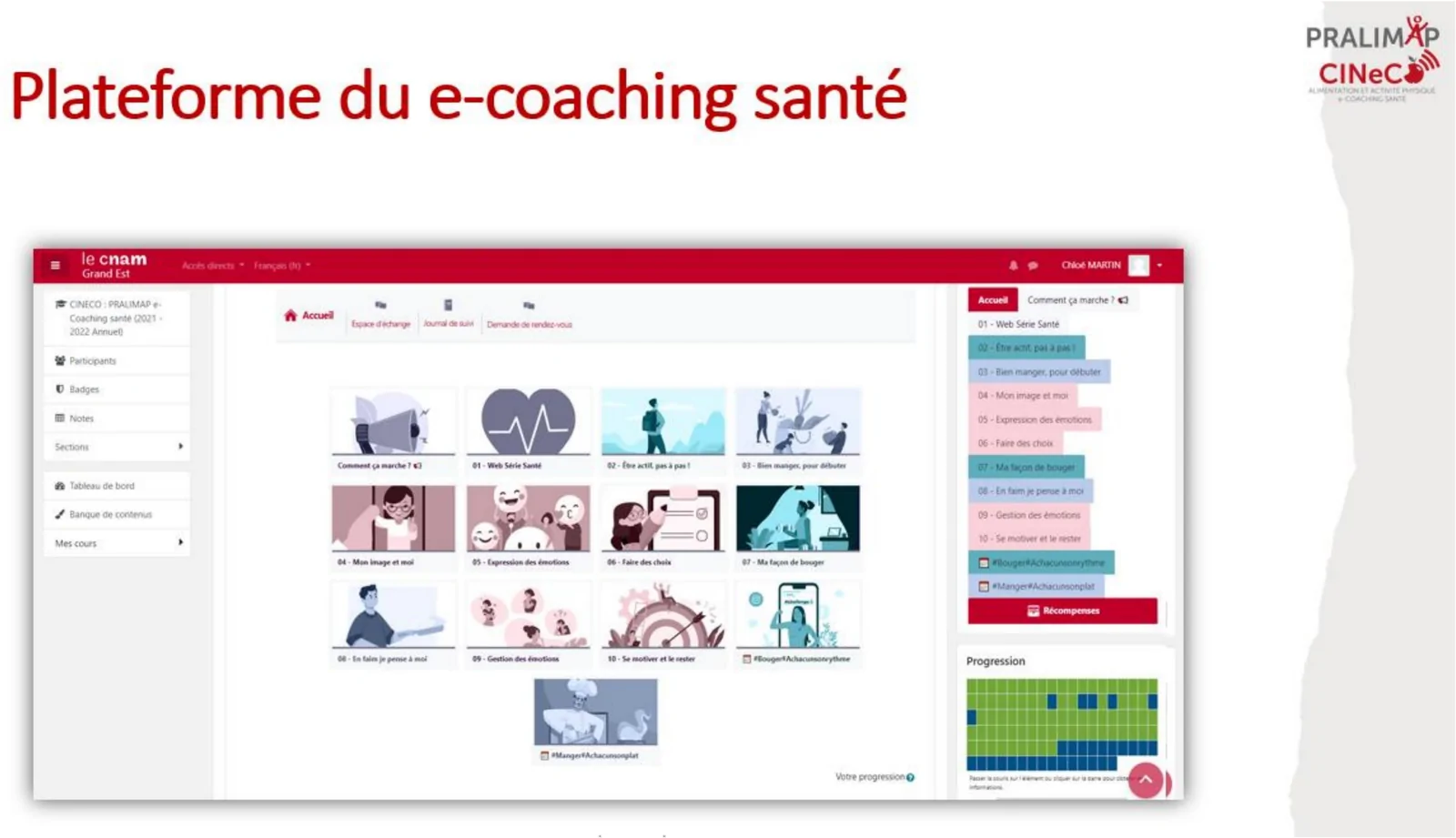
The online health e-coaching platform.

The health e-coaching intervention was developed through a multistep process. Input from professionals (including psychologists, sociologists, and obesity specialists, as well as therapeutic education and digital experts) emphasized the importance of personalization, consideration of the social context, collaborative activities, participant choice, and meaningful communication. A focus group was conducted in October 2019 with six former PRALIMAP-INÈS participants and was guided by facilitators from the research team. Feedback on their experiences and expectations was used to refine the e-coaching design. Content was developed by using the ADDIE instructional design model (Analysis, Design, Development, Implementation, and Evaluation). Various tools and media (including film production and the creation of animated content in a variety of formats, such as instructional, training or step-by-step guides), as well as interactive content creation tools (such as Prezi, Genially and Canva), were employed to ensure engaging, varied, and interactive activities.

A full description of the HeC intervention is presented in the **Supplementary Material 1**.

#### 2.3.2 Standard care

Participants in the standard care group were invited to consult a health care professional (specifically involving their general practitioner) to discuss the results of their medical visit. No intervention was planned for the participants in this group. However, participants were free to consult any health care professional of their choice, as they would not be involved in the study. As the follow-up visit was scheduled for 6 to 12 months after inclusion (T1), a telephone reminder system was implemented to maintain participant engagement. A physician contacted each participant in the SC group at 1 to 2 months after T0 to confirm receipt of the summary letter. During this call, the physician reminded them about the study, reviewed any health-related steps that they had enacted, and reiterated the upcoming T1 visit.

### 2.4 Measurements

#### 2.4.1 Primary outcome

The primary outcome was a binary (yes/no) composite of improvement from T0 to T1 in at least two of the following three components: (1) eating behavior; (2) physical activity and sedentary behavior; and (3) eating self-regulation. Therefore, the primary outcome was considered achieved when improvement from T0 to T1 was observed in components (1) and (2), or (1) and (3), or (2) and (3).

– Eating behavior component: Eating behavior was assessed by using a French food frequency questionnaire known as the “Boire, Manger, Bouger” (Arrighi et al., 2009). This questionnaire allows for the frequencies of consumption per week of 10 food groups to be calculated, including cereal; starchy foods; dairy products; fruits; vegetables; meats and eggs; fish; sugary products; sugary beverages; and snacking. For each food group, improvement was defined as an increase or a decrease of at least one serving per week (depending on the specific food group) based on the recommendation of the French National Nutrition and Health Program (Ministère de la Santé, des Familles, de l'Autonomie, et des Personnes handicapées, 2026) (**Supplementary Table 1**).The eating behavior component was considered improved when the frequency of consumption improved for at least half of the assessed food groups (i.e., at least 5 of 10 groups) from T0 to T1; otherwise, it was considered not improved.
– Physical activity and sedentary behavior component: Physical activity and sedentary behavior were measured by using the French version of the Global Physical Activity Questionnaire, which exhibits acceptable reliability (Kappa = 0.50–0.62) and validity (Spearman *r* = 0.41–0.86) in an adult French population (Rivière et al., 2018). The questionnaire includes 16 items designed to assess the frequency and duration of physical activity across three domains (work, leisure time, and transportation) and one sedentary behavior domain (time spent sitting during a typical week). It enables the calculation of weekly physical activity duration (min/week) for each domain, and total sitting time (minutes per day). For physical activity, improvement was defined as an increase of at least 30 min/week in each domain based on World Health Organization guidelines (Bull et al., 2020) (**Supplementary Table 1**). With respect to total sitting time, improvement was defined as a decrease of at least 60 min per day (Bull et al., 2020) (**Supplementary Table 1**). The physical activity and sedentary behavior component was considered improved when the duration improved for at least 2 of the 4 physical activity and sedentary behavior domains (i.e., work, leisure time, transportation, and total sitting time) from T0 to T1; otherwise, it was considered not improved.
– Eating self-regulation component: Eating self-regulation was assessed by using the validated (Cronbach's alpha = 0.73–0.78) Tempest Self-Regulation Questionnaire for Eating, which consists of 24 items scored on a 5-point Likert scale (from “never” to “always”) (De Vet et al., 2014). The questionnaire allows for the measurement of 6 self-regulation strategies. Two strategies address temptations (including avoidance of temptations and controlling temptations), two strategies address the meaning of temptations (such as distraction and suppression), and two strategies address the goal (including goal and rule setting, as well as goal deliberation). The scores of each strategy were normalized from 0 to 100. Higher scores indicate more frequent use of self-regulation strategies. In the absence of a minimum clinically important difference threshold for each eating self-regulation strategy, a score increase of at least 10 points (i.e., 10%) was defined as improvement based on the clinically meaningful magnitude of change for patient-reported outcome (Ringash et al., 2007) (**Supplementary Table 1**). The eating-self-regulation component was considered improved when the score improved for at least 3 out of 6 eating self-regulation strategies from T0 to T1; otherwise, it was considered not improved.

#### 2.4.2 Secondary outcomes

The secondary outcomes included changes in eating behavior, physical activity, sedentary behavior, eating self-regulation, BMI (kg/m^2^), abdominal circumference (cm), eating disorders, anxiety and depression, and QoL from T0 to T1.

Eating behavior secondary outcomes included changes in the frequency of consumption of each food group (serving/week); the total number of food groups improved; and improvement in the eating behavior component (yes/no). Physical activity and sedentary behavior secondary outcomes included changes in the duration of each physical activity domains (min/week), total physical activity duration (i.e., the sum of the three physical activity domains) (min/week), and total sitting time duration (min/day), as well as the total number of physical activity and sedentary behavior domains improved, and improvement in the physical activity and sedentary behavior component (yes/no). Eating self-regulation secondary outcomes included changes in each eating self-regulation strategy score, the total number of eating self-regulation strategies improved, and improvement in the eating self-regulation component (yes/no). BMI was calculated from height in m (measured by using a measuring rod) and weight in kg (measured by using a balance) during the medical visits. Abdominal circumference was measured by using a tape measure during the medical visits. The Eating Attitudes Test was used to assess eating disorders (Haines et al., 2011). This test is a validated (Cronbach's alpha = 0.86) 26-item self-report questionnaire with responses scored on a 7-point Likert scale from 0 (never) to 6 (always); moreover, it provides a score for dieting, bulimia, and oral control, as well as a total score (Leichner et al., 1994). Anxiety and depression were measured by using the Hospital Anxiety and Depression Scale (Untas et al., 2009), which is a validated (Cronbach's alpha = 0.82 for depression and 0.79 for anxiety) 14-item self-report questionnaire involving 14 items scored on a 4-point Likert scale. This questionnaire provides a score for anxiety and a score for depression. QoL was assessed by using the 12-Item Short Form Health Survey (SF-12), which is a validated (correlation coefficient with SF-36 = 0.94–0.97) self-report questionnaire including 12 items providing two composite scores: the Physical Component Summary (PCS) and the Mental Component Summary (MCS) (Gandek et al., 1998). Scores were normalized to range from 0 to 100, with 0 representing the lowest levels of eating disorders, anxiety and depression symptoms, and QoL.

#### 2.4.3 Sociodemographic characteristics

Sociodemographic characteristics were self-reported by young adults at T0 and consisted of sex (male, female), age (years), educational level (≤Bachelor's degree; >Bachelor's degree and <Master's degree; or Master's degree/PhD), monthly income (<1500€; ≥1500€ and < 2800€; and ≥2800€), single (yes or no), and perceiving this relationship as being a long-term relationship (yes or no).

Except for sociodemographic characteristics, which were collected only at T0, all data were collected at both T0 and T1, either through an online self-administered questionnaire (i.e., eating behavior, physical activity, sedentary behavior, eating self-regulation, eating disorders, anxiety and depression, and QoL) or during the medical visit (i.e., BMI, and abdominal circumference).

### 2.5 Sample size

The initial number of subjects required for this study was estimated based on the assumption that we would be able to recontact 50% of the PRALIMAP-INÈS former participants (n = 852), along with the assumptions that 50% of these participants would meet the inclusion criteria and 10% would refuse to participate. This design provides an estimated 384 young adults to be included (192 in each group). Under these conditions, with a power of 80% and a unilateral alpha risk of 5%, the minimum differences that could be detected were estimated to range from 7.6% to 14.2% for the primary outcome (depending on the expected proportion in the SC group). Given the difficulties associated with the inclusion of former PRALIMAP-INÈS participants, we opened enrollment in the PRALIMAP-CINeCO study to students at the University of Lorraine who volunteered to participate (Fig. 2).

**Fig. 2:**
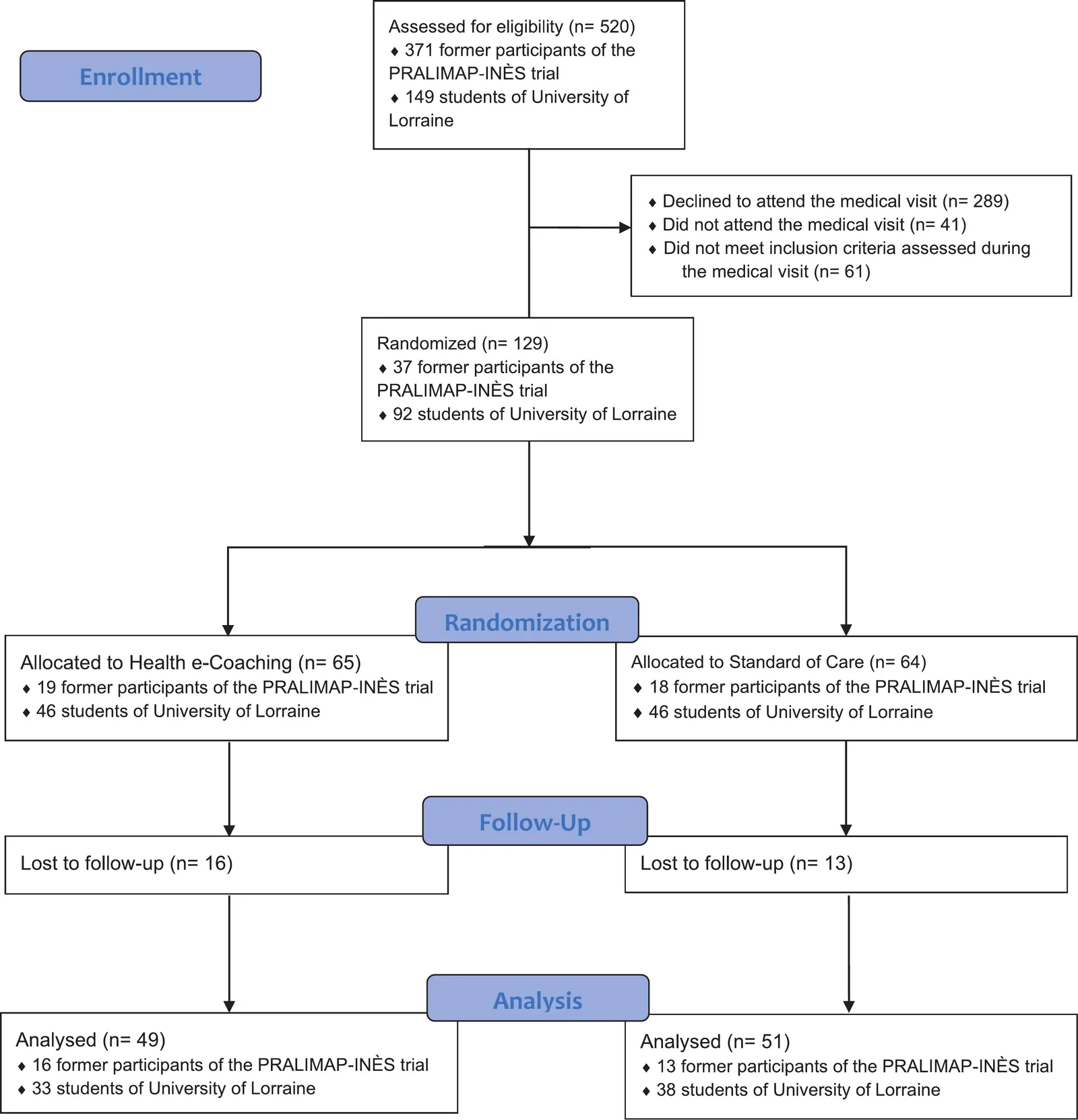
CONSORT flowchart of the PRALIMAP-CINECO trial.

### 2.6 Randomization

Randomization by minimization was performed by a biostatistician on the research team and was stratified based on the initial PRALIMAP-INÈS intervention group. The unit of randomization included young adults. Young adults were randomized into either the HeC (i.e., intervention) group or the SC (i.e., control) group during a period of 12 months at a 1:1 allocation ratio. Due to its nature, participants could not be blinded to the intervention.

### 2.7 Statistical analysis

The overall baseline sociodemographic characteristics of the participants are described; specifically, according to the intervention group, they are described by using means ± standard deviations for continuous variables and as numbers and percentages for categorical variables. Missing data were reported. The sociodemographic characteristics of the participants who were lost to follow-up or not were compared by using the chi-square test or Fisher's exact test for categorical variables and the Student's *t*-test for continuous variables.

The effectiveness of the HeC versus the SC intervention was evaluated by comparing differences in the means (for continuous outcomes) or percentages (for categorical outcomes) between the two groups from T0 to T1. For binary categorical outcomes, including the primary outcome, outcome status (yes/no) was used as the response variable, with condition (HeC vs. SC) included as the explanatory variable in logistic regression models. For continuous outcomes, which were all secondary outcomes, change scores from T0 to T1 were used as the response variables, with condition (HeC vs. SC) included as the explanatory variable in linear regression models. Analyses were performed using a complete-case approach, including participants with available outcome data at T0 and T1, who were analyzed according to their original randomized group allocation. No imputation of missing outcome data was performed. The analyses were not adjusted for sociodemographic characteristics due to randomization. Regression coefficients (β) or odds ratios (ORs) and 95% confidence intervals (CIs) are reported.

A value of *p* < 0.05 was considered to indicate statistical significance. Statistical analyses were performed by using SAS 9.4™ software (SAS v9.34, SAS Inst., Cary, NC, USA).

### 2.8 Ethical considerations

The PRALIMAP-CINeCO trial has been approved by the French Persons Protection Committee (no. 2021-A02425-36), and a conformity declaration with the French National Commission for Data Protection and Liberties has been made. This trial was registered at ClinicalTrials.gov (NCT05399407).

## 3 Results

### 3.1 Study sample

The flowchart of the study is shown in Fig. 2. A total of 520 young adults were eligible for the study. Among them, 391 were not included, mainly due to the fact that they did not want to attend the medical visit (n = 289). A total of 129 young adults were randomized at T0 (n = 65 in the HeC group and n = 64 in the SC group). Twenty-nine (n = 16 in the HeC group and n = 13 in the SC group) young adults were lost to follow-up, thus resulting in 100 young adults who participated at T1 (n = 49 in the HeC group and n = 51 in the SC group). Young adults who were lost to follow-up were more likely to be single than in a relationship (**Supplementary Table 2**).

### 3.2 Sociodemographic characteristics

The characteristics of the participants who completed the study are described in Table 1. The mean age was 24.5 ± 5.1 years. Moreover, a total of 69% of the participants were women, 45% had a bachelor's degree or less, and 55% had a monthly income of less than 1500€. Furthermore, the mean BMI was 31.5 ± 5.2 kg/m^2^.

**Table 1:** Description of baseline characteristics of young adults both overall and according to the intervention group (n = 100).

	Overall		Standard care		Health e-coaching	
	n = 100		n = 51		n = 49	
	n	%	n	%	n	%
Sociodemographic characteristics						
Sex						
Male	31	31.0	18	35.3	13	26.5
Female	69	69.0	33	64.7	36	73.5
Age (mean ± SD)	24.5	±5.1	24.4	±4.4	24.6	±5.7
Educational level						
≤Bachelor's degree	45	45.0	22	43.1	23	46.9
>Bachelor's degree and <Master's degree	40	40.0	22	43.1	18	36.7
Master's degree/PhD	15	15.0	7	13.7	8	16.3
Monthly income						
<1500€	55	55.0	28	54.9	27	55.1
≥1500€ and <2800€	19	19.0	10	19.6	9	18.4
≥2800€	18	18.0	9	17.6	9	18.4
Does not know or does not wish to answer	8	8.0	4	7.8	4	8.2
Single						
No	43	43.0	23	45.1	20	40.8
Yes	57	57.0	28	54.9	29	59.2
Perceive the relationship as lasting?						
No	2	3.5	1	3.6%	1	3.4
Yes	55	96.5	27	96.4%	28	96.6
Dependent children						
No	91	94.8	46	95.8	2	4.2
Yes	5	5.2	45	93.7	3	6.3
Anthropometric characteristics						
BMI (mean ± SD)	31.5	±5.2	32.5	±5.2	30.4	±5.2
Abdominal circumference (mean ± SD)	95.4	±14.8	97.9	±16.7	92.9	±12.4

Data are presented as numbers and percentages unless otherwise indicated. SD, standard deviation.

### 3.3 Primary outcome

In the HeC group, 46.5% of the young adults exhibited improvements in at least two of the three components of the primary outcomes compared with 21.4% in the SC group (Table 2). This result translated to a 3.2 times (95% CI [1.2, 8.2]) greater likelihood of improving at least two of the three components of the primary outcome in the HeC group than in the SC group. Moreover, these results tended to be related to improvements in food group frequency consumption and sedentary behavior rather than physical activity and self-regulation strategies. Young adults in the HeC group were 4.3 times (95% CI [1.6, 11.9]) more likely to improve at least half of the food group frequency consumption than the young adults in the SC group (HeC group = 46.5%; SC group = 16.7%). Although statistically nonsignificant, young adults in the HeC group were 2.5 times (95% CI [1.0, 6.3]) more likely to improve at least half of the physical activity and sedentary behavior domains than young adults in the SC group (HeC group = 71.1%; SC group = 50.0%).

**Table 2:** Effectiveness of the health-e-coaching intervention compared to standard care on the primary and secondary outcomes.

	Group		Comparison	
	Standard care	Health e-coaching		
	n = 51	n = 49	β/OR [95% CI]	*p*
Primary outcome: Improvement in at least 2 of 3 components				**0.01**
No	33 (78.6%)	23 (53.5%)	1	
Yes	9 (21.4%)	20 (46.5%)	**3.2 [1.2; 8.2]**	
Secondary outcomes: Total number of components improved				
N/missing	42/9	43/6		
Mean [95% CI]	7.9 [6.9; 8.9]	9.1 [8.1; 10.1]	+1.2 [−0.2; 2.6]	0.10
Number of food groups improved				**0.01**
N/missing	42/9	43/6	**0**	
Mean [95% CI]	4.4 [3.9; 4.8]	5.2 [4.7; 5.7]	**+0.9 [0.2; 1.5]**	
Improvement in at least half of the food groups				**0.003**
No	35 (83.3%)	23 (53.5%)	**1**	
Yes	7 (16.7%)	20 (46.5%)	**4.3 [1.6; 11.9]**	
Number of physical activity and sedentary behavior domains improved				0.06
N/Missing	40/11	38/11		
Mean [95% CI]	1.9 [1.5; 2.4]	2.6 [2.1; 3.0]	+0.6 [−0.0; 1.3]	
Improvement in at least half of the physical activity and sedentary behavior domains				0.06
No	20 (50.0%)	11 (28.9%)	1	
Yes	20 (50.0%)	27 (71.1%)	2.5 [1.0; 6.3]	
Number of eating self-regulation strategies improved				0.54
N/missing	33/18	37/12	0	
Mean [95% CI]	2.2 [1.6; 2.8]	1.9 [1.3; 2.5]	−0.3 [−1.1; 0.6]	
Improvement in at least half of the eating self-regulation strategies				0.71
No	20 (60.6%)	24 (64.9%)	1	
Yes	13 (39.4%)	13 (35.1%)	0.8 [0.3; 2.2]	

β: linear regression model; OR: odds ratio; CI: confidence interval. Statistically significant results (p<0.05) are shown in bold.

### 3.4 Secondary outcomes

#### 3.4.1 Eating behavior

Among the food groups, those individuals that demonstrated more favorable changes in the HeC group compared to the SC group included starchy foods, fish, sugary products, fruits and vegetables (Table 3). For example, the frequency of fruit and vegetable consumption increased by an average of 3.2 servings per week in the HeC group but decreased by −1.7 servings in the SC group, thus resulting in a higher number of 5 servings (95% CI [0.8, 9.1]) in favor of the HeC group.

**Table 3:** Effectiveness of the health e-coaching intervention compared to standard care on eating behavior.

	Group		Comparison	
	Standard care	Health e-coaching		
	n = 51	n = 49	β/OR [95% CI]	*p*
**Cereals** (serving/week)				0.81
T0				
N/missing	51/0	48/1		
Mean ± SD	7.5 ± 6.6	7.1 ± 6.0		
Change: T1 − T0				0.88
N/missing	39/12	41/8	0	
Mean [95% CI]	−1.3 [−3.4; 0.7]	−1.1 [−3.1; 0.8]	0.2 [−2.6; 3.0]	
Improvement: T1 − T0 ≥ 1				0.69
No	25 (64.1%)	28 (68.3%)	1	
Yes	14 (35.9%)	13 (31.7%)	0.8 [0.3; 2.1]	
**Starchy foods** (serving/week)				0.24
T0				
N/Missing	50/1	48/1		
Mean ± SD	10.9 ± 5.2	9.7 ± 4.7		
Change: T1 − T0				0.15
N/Missing	41/10	41/8	0	
Mean [95% CI]	−1.6 [−3.7; 0.5]	0.6 [−1.5; 2.6]	2.2 [−0.8; 5.1]	
Improvement: T1 − T0 ≥ 1				**0.01**
No	36 (87.8%)	27 (65.9%)	**1**	
Yes	5 (12.2%)	14 (34.1%)	**3.7 [1.2; 11.6]**	
**Dairy products** (serving/week)				0.86
T0				
N/missing	51/0	48/1		
Mean ± SD	9.6 ± 6.8	9.8 ± 6.4		
Change: T1 − T0				0.95
N/missing	41/10	42/7	0	
Mean [95% CI]	−0.8 [−3.0; 1.5]	−0.7 [−2.9; 1.6]	0.1 [−3.1; 3.3]	
Improvement: T1 − T0 ≥ 1				0.85
No	31 (75.6%)	31 (73.8%)	1	
Yes	10 (24.4%)	11 (26.2%)	1.1 [0.4; 3.0]	
**Fruits** (serving/week)				0.87
T0				
N/missing	50/1	48/1		
Mean ± SD	7.3 ± 7.0	7.5 ± 7.2		
Change: T1 − T0				0.13
N/missing	39/12	39/10	0	
Mean [95% CI]	−1.3 [−3.1; 0.5]	0.7 [−1.1; 2.5]	2.0 [−0.6; 4.6]	
Improvement: T1 − T0 ≥ 1				0.09
No	29 (74.4%)	22 (56.4%)	1	
Yes	10 (25.6%)	17 (43.6%)	2.2 [0.9; 5.8]	
**Vegetables** (serving/week)				**0.01**
T0				
N/missing	51/0	49/0		
Mean ± SD	9.7 ± 5.0	7.1 ± 5.6		
Change: T1 − T0				**0.02**
N/missing	42/9	42/7	**0**	
Mean [95% CI]	−0.3 [−2.2; 1.7]	**3.0 [1.0; 4.9]**	**3.2 [0.5; 6.0]**	
Improvement: T1 − T0 ≥ 1				**0.02**
No	29 (69.0%)	19 (45.2%)	**1**	
Yes	13 (31.0%)	23 (54.8%)	**2.7 [1.1; 6.6]**	
**Meats and eggs** (serving/week)				0.21
T0				
N/missing	51/0	49/0		
Mean ± SD	8.7 ± 6.1	7.2 ± 6.0		
Change: T1 − T0				0.28
N/missing	42/9	42/7	0	
Mean [95% CI]	−1.6 [−3.6; 0.4]	−0.0 [−2.1; 2.1]	1.6 [−1.3; 4.4]	
Improvement: T1 − T0 ≤ 1				0.62
No	11 (26.2%)	13 (31.0%)	1	
Yes	31 (73.8%)	29 (69.0%)	0.8 [0.3; 2.0]	
**Fish** (serving/week)				0.68
T0				
N/missing	51/0	48/1		
Mean ± SD	0.9 ± 1.0	0.9 ± 1.0		
Change: T1 − T0				**0.02**
N/missing	41/10	41/8	**0**	
Mean [95% CI]	−0.1 [−0.4; 0.1]	**0.3 [0.0; 0.5]**	**0.4 [0.1; 0.8]**	
Improvement: T1 − T0 ≥ 1				**0.03**
No	39 (95.1%)	33 (80.5%)	**1**	
Yes	2 (4.9%)	8 (19.5%)	**4.7 [1.0; 23.8]**	
**Sugary products** (serving/week)				0.69
T0				
N/missing	51/0	49/0		
Mean ± SD	8.0 ± 6.9	7.4 ± 5.9		
Change: T1 − T0				0.32
N/missing	41/10	42/7	0	
Mean [95% CI]	−1.5 [−3.8; 0.7]	**−3.1 [−5.3; −0.9]**	−1.6 [−4.7; 1.6]	
Improvement: T1 − T0 ≤ 1				**0.02**
No	12 (29.3%)	4 (9.5%)	**1**	
Yes	29 (70.7%)	38 (90.5%)	**3.9 [1.1; 13.5]**	
**Sugary beverages** (serving/week)				0.89
T0				
N/missing	51/0	48/1		
Mean ± SD	3.5 ± 5.9	3.7 ± 5.3		
Change: T1 − T0				0.43
N/missing	40/11	40/9	0	
Mean [95% CI]	−0.3 [−1.7; 1.0]	−1.1 [−2.4; 0.2]	−0.7 [−2.6; 1.1]	
Improvement: T1 − T0 ≤ 1				0.76
No	6 (15.0%)	7 (17.5%)	1	
Yes	34 (85.0%)	33 (82.5%)	0.8 [0.3; 2.7]	
**Snacking** (serving/week)				0.20
T0				
N/missing	51/0	48/1		
Mean ± SD	2.2 ± 1.9	1.8 ± 1.5		
Change: T1 − T0				0.27
N/missing	41/10	40/9	0	
Mean [95% CI]	**−0.8 [−1.4; −0.3]**	−0.4 [−0.9; 0.2]	0.4 [−0.4; 1.2]	
Improvement: T1 − T0 ≤ 1				0.14
No	6 (14.6%)	2 (5.0%)	1	
Yes	35 (85.4%)	38 (95.0%)	3.3 [0.6; 17.2]	
**Fruits and vegetables** (serving/week)				0.27
T0				
N/missing	51/0	49/0		
Mean ± SD	16.8 ± 10.6	14.4 ± 11.3		
Change: T1 − T0				**0.02**
N/missing	42/9	42/7	**0**	
Mean [95% CI]	−1.7 [−4.7; 1.2]	**3.2 [0.3; 6.2]**	**5.0 [0.8; 9.1]**	
Improvement: T1 − T0 ≥ 1				**0.01**
No	27 (64.3%)	16 (38.1%)	**1**	
Yes	15 (35.7%)	26 (61.9%)	**2.9 [1.2; 7.1]**	
**Sugary products and beverages** (serving/week)				0.82
T0				
N/missing	51/0	49/0		
Mean ± SD	11.5 ± 10.2	11.0 ± 8.9		
Change: T1 − T0				0.25
N/missing	41/10	42/7	0	
Mean [95% CI]	−1.9 [−4.6; 0.8]	**−4.1 [−6.8; −1.6]**	−2.2 [−6.1; 1.6]	
Improvement: T1 − T0 ≤ 1				0.39
No	11 (26.8%)	8 (19.0%)	1	
Yes	30 (73.2%)	34 (81.0%)	1.6 [0.6; 4.4]	

β: linear regression model; OR: odds ratio; CI: confidence interval. Statistically significant results (p< 0.05) are shown in bold.

#### 3.4.2 Physical activity and sedentary behavior

There was no significant difference in the change in physical activity outcomes between the HeC group and the SC group (Table 4). The total sitting time per week significantly decreased by 91.6 min/day in the HeC group, which demonstrated a 3.7 (95% CI [1.0, 14.3]) greater likelihood of decreasing total sitting time in comparison with the SC group.

**Table 4:** Effectiveness of the health e-coaching intervention compared to standard care on physical activity and sedentary behavior.

	Group		Comparison	*p*
	Standard care	Health e-coaching		
	n = 51	n = 49	β/OR [95% CI]	
**Physical activity at work (min/week)**				
T0				0.75
N/Missing	51/0	49/0		
Mean ± SD	136.1 ± 333.3	121.2 ± 431.5		
Change: T1 − T0				0.75
N/missing	40/11	38/11	0	
Mean [95% CI]	66.5 [−122.2; 255.2]	109.7 [−83.9; 303.4]	43.2 [−227.2; 313.7]	
Improvement: T1 − T0 ≥ 30				0.51
No	34 (85.0%)	30 (78.9%)	1	
Yes	6 (15.0%)	8 (21.1%)	1.5 [0.5; 4.6]	
**Physical activity during leisure time (min/week)**				
T0				0.45
N/missing	51/0	48/1		
Mean ± SD	179.2 ± 287.0	140.6 ± 206.3		
Change: T1 − T0				0.38
N/Missing	40/11	36/13	0	
Mean [95% CI]	−4.3 [−95.5; 87.0]	54.9 [−41.3; 151.0]	59.1 [−73.4; 195.7]	
Improvement: T1 − T0 ≥ 30				0.38
No	24 (60.0%)	18 (50.0%)	1	
Yes	16 (40.0%)	18 (50.0%)	1.4 [0.6; 3.1]	
**Transportation (min/week)**				
T0				0.53
N/Missing	49/2	46/3		
Mean ± SD	171.1 ± 173.2	147.8 ± 189.2		
Change: T1 − T0				0.35
N/Missing	37/14	35/14	0	
Mean [95% CI]	12.6 [−30.9; 56.1]	41.9 [−2.9; 86.6]	−29.3 [−33.1; 91.7]	
Improvement: T1 − T0 ≥ 30				0.10
No	24 (64.9%)	16 (45.7%)	1	
Yes	13 (35.1%)	19 (54.3%)	1.9 [0.8; 4.4]	
**Total physical activity (min/week**)				
T0				0.51
N/Missing	48/3	45/4		
Mean ± SD	469.4 ± 438.0	402.4 ± 533.6		
Change: T1 − T0				0.53
N/Missing	33/18	33/16	0	
Mean [95% CI]	100.6 [−154.0; 355.2]	214.2 [−40.3; 468.8]	113.6 [−246.4; 473.7]	
Improvement: T1 − T0 ≥ 60				0.21
No	19 (57.6%)	14 (42.4%)	1	
Yes	14 (42.4%)	19 (57.6%)	1.7 [0.7; 4.1]	
**Total sitting time (min/day)**				
T0				0.53
N/Missing	51/0	49/0		
Mean ± SD	576.2 ± 186.4	599.4 ± 182.0		
Change: T1 − T0				0.06
N/Missing	39/12	37/12	0	
Mean [95% CI]	−18.1 [−72.4; 36.3]	**−91.6 [−147.4; −35.8]**	−73.5 [−151.4; 4.3]	
Improvement: T1 − T0 ≤ 60				**0.04**
No	10 (25.6%)	3 (8.1%)	**1**	
Yes	29 (74.4%)	34 (91.9%)	**3.7 [1.0; 14.3]**	

β: linear regression model; OR: odds ratio; CI: confidence interval. Statistically significant results (p< 0.05) are shown in bold.

#### 3.4.3 Eating self-regulation

There was no evidence of statistical difference in the change in eating self-regulation strategies between the HeC group and the SC group (Table 5).

**Table 5:** Effectiveness of the health e-coaching intervention compared to standard care on eating self-regulation.

	Group		Comparison	*p*
	Standard care	Health e-coaching		
	n = 51	n = 49	β/OR [95% CI]	
**Avoidance of temptations** (0−100)				
T0				0.53
N/missing	41/10	47/2		
Mean ± SD	25.3 ± 19.8	28.0 ± 21.2		
Change: T1 − T0				0.50
N/missing	33/18	36/13	0	
Mean [95% CI]	4.3 [−2.6; 11.2]	1.1 [−5.5; 7.7]	−3.2 [−12.7; 6.3]	
Improvement: T1 − T0 ≥ 10				0.83
No	24 (72.7%)	27 (75.0%)	1	
Yes	9 (27.3%)	9 (25.0%)	0.9 [0.3; 2.6]	
**Controlling temptations** (0–100)				
T0				0.90
N/missing	42/9	48/1		
Mean ± SD	34.3 ± 26.5	34.9 ± 22.7		
Change: T1 − T0				0.87
N/missing	33/18	37/12	0	
Mean [95% CI]	2.1 [−5.7; 10.0]	1.3 [−6.1; 8.7]	−0.8 [−11.6; 9.9]	
Improvement: T1 − T0 ≥ 10				0.72
No	21 (63.6%)	22 (59.5%)	1	
Yes	12 (36.4%)	15 (40.5%)	1.2 [0.5; 3.1]	
**Distraction** (0–100)				
T0				0.51
N/missing	41/10	48/1		
Mean ± SD	29.6 ± 19.9	32.2 ± 17.7		
Change: T1 − T0				0.36
N/missing	33/18	37/12	0	
Mean [95% CI]	5.0 [−2.0; 12.1]	0.7 [−5.9; 7.3]	−4.4 [−14.0; 5.3]	
Improvement: T1 − T0 ≥ 10				0.27
No	20 (60.6%)	27 (73.0%)	1	
Yes	13 (39.4%)	10 (27.0%)	0.6 [0.2; 1.6]	
**Suppression** (0–100)				
T0				0.18
N/missing	41/10	47/2		
Mean ± SD	29.6 ± 18.4	34.9 ± 18.6		
Change: T1 − T0				0.30
N/missing	33/18	36/13	0	
Mean [95% CI]	4.0 [−4.4; 12.3]	−2.1 [−10.2; 5.9]	−6.2 [−17.7;5.5]	
Improvement: T1 − T0 ≥ 10				0.60
No	21 (63.6%)	25 (69.4%)	1	
Yes	12 (36.4%)	11 (30.6%)	0.8 [0.3; 2.1]	
**Goal and rule setting** (0–100)				
T0				0.70
N/missing	42/9	47/2		
Mean ± SD	30.2 ± 22.7	32.0 ± 21.0		
Change: T1 − T0				0.95
N/missing	33/18	36/13	0	
Mean [95% CI]	**8.1 [1.1; 15.1]**	**8.4 [1.7; 15.1]**	0.3 [−9.4; 10.0]	
Improvement: T1 − T0 ≥ 10				0.36
No	21 (63.6%)	19 (52.8%)	1	
Yes	12 (36.4%)	17 (47.2%)	1.6 [0.6; 4.1]	
**Goal deliberation** (0–100)				
T0				0.24
N/missing	42/9	48/1		
Mean ± SD	39.9 ± 22.6	44.9 ± 17.5		
Change: T1 − T0				0.25
N/missing	33/18	37/12	0	
Mean [95% CI]	6.1 [−2.5; 14.6]	−0.8 [−8.9; 7.3]	−6.8 [−18.6; 4.9]	
Improvement: T1 − T0 ≥ 10				0.10
No	19 (57.6%)	28 (75.7%)	1	
Yes	14 (42.4%)	9 (24.3%)	0.4 [0.2; 1.2]	

β: linear regression model; OR: odds ratio; CI: confidence interval. Statistically significant results (p< 0.05) are shown in bold.

#### 3.4.4 BMI and abdominal circumference

Although BMI did not significantly change in the HeC group, it decreased to a greater degree in the SC group by 1.2 points (95% CI [0.3, 2.1]). The trend was similar for abdominal circumference (**Supplementary Table 3**).

#### 3.4.5 Eating disorders, anxiety, depression, and QoL

No significant change was observed between the two groups in terms of eating disorders, anxiety, depression and PCS, whereas the change in the MCS score tended to increase to a greater degree by 3.8 points (95% CI [−0.2, 7.7]) in the HeC group than in the SC group (**Supplementary Tables 4–6**).

## 4 Discussion

### 4.1 Principal findings

The PRALIMAP-CINeCO trial provides evidence for the effectiveness of a personalized HeC intervention (combining asynchronous online individual activities and regular teleconsultations with health professionals) compared with SC in young adults who are with overweight or obesity. An improvement in a combination of eating behavior, physical activity, sedentary behavior, and eating self-regulation outcomes was 3.2 times more common in the HeC group than in the SC group. This effectiveness tended to be more strongly related to improvements in food group frequency consumption and sedentary behavior than to improvements in physical activity and eating self-regulation strategies.

The effectiveness observed on the primary outcome should be interpreted in light of the multicomponent nature of the HeC intervention, which intentionally combined asynchronous online activities with personalized support from health professionals based on existing evidence (Kupila et al., 2023). These components were designed to be complementary, and the study was not intended to disentangle their respective contributions. Consequently, it is not possible to attribute the observed effects to either component specifically. Nevertheless, this multifaceted approach could offer an alternative or complement to conventional face-to-face therapeutic patient education programs, particularly for individuals facing barriers to accessing healthcare services or reluctant to participate in group-based sessions. It could also help reinforce knowledge and skills acquired through previous educational interventions or serve as an initial step towards engagement in conventional therapeutic patient education programs. However, while this approach has the potential to improve accessibility for some populations, its contribution to health inequalities and long-term sustainability remains to be demonstrated. The next challenge is therefore to understand how to implement the intervention in real-world settings and ensure its sustainability over time. The implementation of complex interventions such as HeC requires the consideration of contextual and organizational factors that may influence adoption, such as digital accessibility and literacy, staff availability, and user engagement. In this context, implementation strategies should seek to preserve fidelity to the intervention's “core components” (i.e., the essential and indispensable elements of the intervention, such as online activities and professional guidance), while allowing adaptations to its “adaptable periphery” (i.e., adaptable elements, structures, and systems related to the intervention and organization into which it is being implemented) to a particular real-world setting (Damschroder et al., 2009). Participatory and codesign approaches (involving both health professionals and target users) could help to adapt the intervention to the context, in order to increase its feasibility and acceptability. Potential target populations for future implementation could include individuals with chronic conditions; adolescents and young adults who are familiar with digital tools; and populations in underserved or remote areas. In these areas, limited access to healthcare services due to medical deserts, reduced availability of affordable healthy food options, and insufficient infrastructure supporting physical activity may exacerbate health inequalities and hinder preventive care engagement. For these populations, e-coaching could contribute to reducing social and territorial health inequalities by providing accessible, continuous, and personalized guidance (Veinot et al., 2018). However, its ability to reach underserved populations equitably, as well as its cost-effectiveness and sustainability, requires further evaluation.

In addition to the statistical significance of our results, their clinical significance must be emphasized. The HeC intervention increased fruit and legume consumption by more than 3 servings/week, thereby increasing from 14 portions/week (i.e., 2 portions/day) to nearly 18 portions/week (i.e., 2.6 portions/day), in addition to decreasing the consumption of sugary products and beverages by 4 servings/week, as well as decreasing the total sitting time by 1.5 h/day. These changes align with World Health Organization recommendations and the French National Nutrition and Health Program, including eating 5 portions of fruit and vegetables per day, reducing sugar intake and limiting the amount of time spent sitting (Ministère de la Santé, des Familles, de l'Autonomie, et des Personnes handicapées, 2026; Bull et al., 2020; World Health Organization, 2025).

Unexpectedly, BMI decreased to a greater degree in the SC group than in the HeC group. One possible explanation for this result is that the mean baseline BMI tended to be higher in the SC group (Lang et al., 2017). As a result, participants in this group may have been more likely to experience greater absolute reductions in BMI during the study compared with those in the HeC group, thus potentially reflecting short-term weight fluctuations or yo-yo dieting (Lang et al., 2017).

The potential lack of effectiveness of the HeC intervention on eating self-regulation strategies could reflect the complexity of self-regulation and both content- and format-related limitations of the HeC intervention (Pierce-Messick and Corbit, 2021). In their review, Werner et al. described self-regulation as relying on a repertoire of strategies (a “toolbox”) that individuals can mobilize to support behavior changes (Werner and Ford, 2023). The capacity to regulate eating behavior depends on the breadth and adaptability of this repertoire, with a larger “toolbox” being associated with greater effectiveness in pursuing goals such as healthy eating (Werner and Ford, 2023). For example, one strategy for regulating eating behavior is to develop healthy eating habits (Mann and Ward, 2025). However, habits take time to form, and certain aspects of eating behavior may make this process particularly challenging, thus often requiring prolonged efforts to achieve significant and lasting changes (Keller et al., 2021). Moreover, in addition to the number of available strategies, their flexibility and contextual appropriateness appear to be critical for effective self-regulation (Mann and Ward, 2025). However, such complexity (requiring both a diverse repertoire and the ability to adapt strategies to specific contexts) could be particularly challenging to fully address within the content and format of digital interventions, especially when they rely on asynchronous components. These skills typically require sustained practice, longer intervention duration, and ongoing reinforcement to achieve lasting change. In this context, digital interventions may be more effective when they are multicomponent in nature and incorporate interactive, supported, or group-based elements (rather than purely asynchronous formats). In addition, engagement (which is partially driven by features such as reminders and adaptive support) could play a central role in intervention effectiveness.

### 4.2 Limitations

The results of this study should be interpreted based on its strengths and limitations. The main strengths of this study include the experimental design and the HeC intervention, which combined both online activities and professional guidance; moreover, the need for such interventions was highlighted (Lau et al., 2020). The main limitation is related to the study sample. Although we aimed to include 384 young adults to determine the difference between the HeC and SC groups, a total of 129 young adults were included in the trial, of whom 100 completed the follow-up assessment at T1. This discrepancy was related to the difficulty in contacting former participants of the PRALIMAP-INÈS trial; however, we extended the study to participants of a university, which allowed us to significantly increase the number of included participants, although it was still lower than estimated. This relatively small number of enrolled young adults and the use of a complete-case analysis may have decreased the power of the study and prevented us from demonstrating the effectiveness of the HeC group compared to the SC group on some outcomes. For example, for the improvement in total physical activity from T0 to T1, assuming an alpha risk of 5% and 80% power, and based on the observed proportions of 42.4% in the SC group and 57.6% in the HeC group, a total sample size of 338 participants (169 per group) would have been required to detect a statistically significant difference between the two groups. With our final sample of 100 participants, the statistical power to detect the observed difference was only 33%. Another limitation is the absence of an evaluation of the intervention dose. Such an evaluation would provide valuable insights into how the intervention was delivered, how participants were exposed to it and its potential relationship with the observed outcomes. However, this was beyond the scope of this article. Future research could address this by building on methodological approach previously developed (Legrand et al., 2012).In addition, young adults who were lost to follow-up during the study were more likely to be in a relationship than single, which could limit the generalizability of our results (given the potential association between relationships and health behaviors) (Nizamani et al., 2024). Furthermore, the questionnaire used to assess eating self-regulation was originally validated in adolescents, and its psychometric properties may not fully generalize to adults, potentially limiting sensitivity to age-specific self-regulatory processes, introducing measurement bias, and limiting us to evidence changes in eating self-regulatory strategies.

## 5 Conclusions

Despite a smaller-than-expected sample size and limited resources, the HeC intervention was proven to be effective for the primary outcome, which appeared to be mainly driven by improvements in eating and sedentary behaviors, thus highlighting the potential use of online activities combined with health professional guidance approaches to improve health behaviors. The limited effect observed on eating self-regulation suggests the persistent challenge of modifying deeply ingrained behaviors. Overall, PRALIMAP-CINeCO illustrates the feasibility of a complex, context-adapted digital intervention, thereby providing valuable insights for the future implementation of scalable e-coaching programs in real-world settings.

## CRediT authorship contribution statement

**Florian Manneville**: Conceptualization, Funding acquisition, Investigation, Methodology, Project administration, Supervision, Validation, Visualization, Writing – original draft; **Johanne Langlois**: Conceptualization, Funding acquisition, Investigation, Supervision, Validation, Visualization, Writing – review & editing; **Philip Böhme**: Conceptualization, Funding acquisition, Methodology, Writing – review & editing; **Mélanie Béguinet**: Conceptualization, Investigation, Project administration, Writing – review & editing; **Karine Legrand**: Conceptualization, Funding acquisition, Investigation, Methodology, Project administration; **Willy Ngueyon Sime**: Data curation, Formal analysis; **Elisabeth Spitz**: Conceptualization, Funding acquisition, Investigation, Methodology, Supervision, Validation, Writing – review & editing; **Serge Briançon**: Conceptualization, Funding acquisition, Methodology, Supervision, Writing – review & editing; **Edith Lecomte**: Conceptualization, Funding acquisition, Methodology, Supervision, Validation, Writing – review & editing; **Abdou Y. Omorou**: Conceptualization, Formal analysis, Funding acquisition, Investigation, Methodology, Project administration, Supervision, Validation, Visualization, Writing – review & editing.

## Funding

This study is funded by the Institut pour la Recherche en Santé Publique (IReSP) (grant number: 18II076).

## Declaration of competing interest

The authors declare that they have no known competing financial interests or personal relationships that could have appeared to influence the work reported in this paper.

## Data Availability

The datasets that were generated or analyzed during this study are available from the corresponding author upon reasonable request.
