# Supplementary material for: Effectiveness of a personalized e-coaching intervention on nutritional behavior and eating self-regulation among adults with overweight or obesity: The PRALIMAP-CINeCO randomized trial
Source: Internet Interv. 2026 Oct 30;46:101019. doi: 10.1016/j.invent.2026.101019 (PMC13742595; doi:10.1016/j.invent.2026.101019)
Supplement: Supplementary file 1 [file mmc1.docx]

**Supplementary Material 1. Detailed description of the health e-coaching intervention**

1. **Health e-coaching development**

The development of health e-coaching required several preliminary steps.

a. ***Needs and expectations***

***Professional perspective:*** Discussions were held with various professionals, including psychologists, sociologists, obesity specialists, therapeutic education professionals, and digital professionals, which helped to highlight certain essential elements in the development of the tool:

- Promote personalized intervention for each participant, particularly for people suffering from obesity.
- Consider the social environment and context in which the participant grew up.
- Encourage collaborative activities
- Give participants a choice as often as possible.
- Deploy meaningful communication

***Young adults' perspective:*** A panel of young adult (n= 13) who were part of the PRALIMAP-INÈS cohort was contacted. Prior to this, a contact guide for the PRALIMAP CINECO focus group call for participation was drafted, including an information collection grid.

The focus group took place in October 2019, with two facilitators and one observer in attendance of the research team, as well as six young adults who had participated in PRALIMAP-INÈS.

An interview guide was prepared in advance, consisting of several sections that were addressed:

- Introduction: presentation, focus group objectives
- Feedback on PRALIMAP-INÈS
- PRALIMAP-CINECO: presentation of the project, including its content and the method envisaged for finding/contacting/motivating young adults.

The results were taken into account in the strategy for contacting young adults and in the development of e-coaching in order to adapt to the needs of the target audience.

b. ***Production of e-coaching content***

The ADDIE (Analysis Design Development Implementation Evaluation) model was used for the instructional design of the health e-coaching. This model involves studying a number of components to guide the development of the participants' learning system and consists of five main stages: analysis, design, development, implementation, and evaluation. All of these phases are managed by a coordinator of the research team. Various educational resources/tools were used to make the activities fun, varied (video, computer graphics, animation, quizzes, exercises, interactive images, etc.) and interactive (advanced office suite, Prezi, Genially, Canva, screencast, filming/editing, etc.).

2. **Finalized health e-coaching intervention**

The research team led and finalized the development of the health e-coaching: an innovative, attractive, and customizable program for the care of patients who are overweight or obese. It aims to develop knowledge, skills, and attitudes based on the principles of patient therapeutic education. It facilitates communication between patients, between patients and professionals, and between professionals for coordinated care. The PRALIMAP CINeCO health e-coaching program offers two forms of patient support (Figure 1):

1. Participation in individual asynchronous educational activities
2. Participation in individual follow-up sessions via videoconference with a dietitian, psychologist, and adapted physical activity instructor

These two forms of support are centralized on an online educational platform available to patients and professionals.

During the training of professionals involved in the health e-coaching program, various tools were provided: an interactive and comprehensive presentation of the program, a detailed presentation of the activities on the platform, a user guide for the platform, a guide for initial and intermediate BEP interviews, and a grid to help define personalized programs.

Each patient participates in a shared educational assessment to define their personalized program. This program can be adjusted during an interim assessment.

Before starting, patients are asked to sign the online user agreement for the digital platform hosting PRALIMAP-CINeCO's health e-coaching. To begin, patients can access the "How does it work?" section. There they will find an interactive flyer (**Figure 1**) presenting the e-coaching program, the user guides the discussion forum for participants, their progress log, and a space to contact a healthcare professional when necessary.

**Figure 1**: Interactive flyer of the health e-coaching program

The individual asynchronous educational activities focus on four main themes: general health, physical activity/sedentary behaviors, eating behavior, and psychological well-being. In total, more than 80 activities have been created, all of which are fun, interactive, and participatory: web series, tutorials, serious games, testimonials, quizzes, and more. The activities have been divided into 12 modules (**Figures 2-3**):

- There is one health module: "Health Web Series."
- There are three modules on physical activity/sedentary lifestyles: "Being active step by step," "My way of moving," and "#Move#AtYourOwnPace."
- There are three modules on nutrition: "Eating well to start with," "When I'm hungry, I think of myself," and "#Eating#EveryoneTheirOwnMeal."
- There are five modules in psychology: "My image and me," "Expressing emotions," "Making choices," "Managing emotions," and "Motivating yourself and staying motivated."

Each of these modules contains between 4 and 15 activities. All activities are accessible with a single click and can be viewed as many times as necessary, in accordance with the personalized program (**Figure 4**).

**Figure 2.** Content of the e-coaching health platform

**Figure 3.** Examples of activities included in the online health e-coaching platform

**Figure 4.** Activities of the health e-coaching program

In addition to monitoring activities, individual support tailored to needs is offered by professionals such as dieticians, adapted physical activity instructors, and psychologists, in the form of regular remote sessions. Several strategies have been developed to maintain patient motivation to participate. Patients can earn virtual badges based on their progress in the health e-coaching program. In total, there are 28 badges that can be earned during the program, depending on participation. Every effort has been made to ensure that patients can easily find their way around the platform with easy navigation: color codes, pictograms, event calendar, visibility of their progress in the e-Health Coaching program.

3. **Key figures**

To set up participant monitoring in health e-coaching, six professionals were trained in the program and involved in patient care: three psychologists, two dietitians, and one adapted physical activity instructor.

In order to adapt to each participant, 60 initial shared educational assessments were carried out with healthcare professionals, with personalized programs co-developed with each patient (60 programs completed).

Each participant had several appointments with professionals, depending on their personalized program: 90 appointments with psychologists for self-management, 52 follow-up appointments with dietitians, and 39 follow-up appointments with adapted physical activity instructors.

In parallel with monitoring by healthcare professionals, asynchronous online activities were organized for participants: 53 took part.

In order to review progress in monitoring e-coaching and reassess any needs/developments, 53 shared interim educational assessments were carried out with healthcare professionals.

4. **Key levers of the health e-coaching program**

- Free of charge for young adults
- Trained and competent professionals to provide remote patient care
- Customized programs, tailored to each young adult's needs and adjustable
- Access to all online activities
- A variety of fun activities focusing on eating behavior, physical activity and sedentary behavior, and psychology
- Follow the content at your own pace
- Follow-up appointments with professionals to support the achievement of objectives

**Supplementary Table 1.** Description of thresholds of improvement of outcomes

| **Outcome** | **Threshold** |
| --- | --- |
| Primary outcome | Improve ≥ 2 out of 3 components |
| Eating behavior | Improve ≥ 5 out of 10 food groups |
| *Cereal* | Increase ≥ 1 serving/week |
| *Starchy food* | Increase ≥ 1 serving/week |
| *Dairy product* | Increase ≥ 1 serving/week |
| *Fruit* | Increase ≥ 1 serving/week |
| *Vegetable* | Increase ≥ 1 serving/week |
| *Fruit and vegetable* | Increase ≥ 1 serving/week |
| *Fish* | Increase ≥ 1 serving/week |
| *Meat and egg* | Decrease ≤ 1 serving/week |
| *Sugary product* | Decrease ≤ 1 serving/week |
| *Sugary beverage* | Decrease ≤ 1 serving/week |
| *Sugary product and beverage* | Decrease ≤ 1 serving/week |
| *Snacking* | Decrease ≤ 1 serving/week |
|  |  |
| Physical activity and sedentary behavior | ≥ 2 out of 4 domains |
| *Physical activity at work* | Increase ≥ 30 minutes/week |
| *Physical activity during leisure-time* | Increase ≥ 30 minutes/week |
| *Transportation* | Increase ≥ 30 minutes/week |
| *Total physical activity* | Increase ≥ 60 minutes/week |
| *Total sitting time* | Decrease ≤ 60 minutes/week |
|  |  |
| Eating self-regulation | ≥ 3 out of 6 strategies |
| *For each strategy* | Increase ≥ 10 points |

**Supplementary Table 2.** Comparison of baseline characteristics between young adults followed-up at T1 and lost to follow-up.

|  |  | | | | **Total** | | **Test** |
| --- | --- | --- | --- | --- | --- | --- | --- |
|  | **Follow-up at T1** | | **Lost to follow-up** | |  |  |  |
|  | **N=100** | | **N=29** | | **N=129** | |  |
| Sociodemographic characteristics |  |  |  |  |  |  |  |
| Gender |  |  |  |  |  |  | Chi-2    p= 0.28 |
| Man | 31 | (31.0%) | 6 | (20.7%) | 37 | (28.7%) |  |
| Women | 69 | (69.0%) | 23 | (79.3%) | 92 | (71.3%) |  |
| Age (mean ± SD) | 24.5 | 5.1 | 23.3 | 3.2 | 24.2 | 4.7 | T-test    p= 0.25 |
| Education level |  |  |  |  |  |  | Chi-2    p= 0.28 |
| ≤ Bachelor’s degree | 45 | (45.0%) | 17 | (58.6%) | 62 | (48.1%) |  |
| >Bachelor’s degree and <Master’s degree | 40 | (40.0%) | 7 | (24.1%) | 47 | (36.4%) |  |
| Master’s degree/ PhD | 15 | (15.0%) | 5 | (17.2%) | 20 | (15.5%) |  |
| Monthly income |  |  |  |  |  |  | Chi-2    p= 0.27 |
| <1500€ | 55 | (55.0%) | 14 | (48.3%) | 69 | (53.5%) |  |
| ≥1500€ and <2800€ | 19 | (19.0%) | 4 | (13.8%) | 23 | (17.8%) |  |
| ≥2800€ | 18 | (18.0%) | 5 | (17.2%) | 23 | (17.8%) |  |
| Does not know or does not wish to answer | 8 | (8.0%) | 6 | (20.7%) | 14 | (10.9%) |  |
| Single |  |  |  |  |  |  | Chi-2    **p= 0.005** |
| No | 43 | (43.0%) | 21 | (72.4%) | 64 | (49.6%) |  |
| Yes | 57 | (57.0%) | 8 | (27.6%) | 65 | (50.4%) |  |
| Perceive the relationship as lasting |  |  |  |  |  |  | Fisher     p= 0.33 |
| No | 2 | (3.5%) | 1 | (12.5%) | 3 | (4.6%) |  |
| Yes | 55 | (96.5%) | 7 | (87.5%) | 62 | (95.4%) |  |
| Dependant children |  |  |  |  |  |  | Fisher     p= 0.58 |
| No | 91 | (94.8%) | 28 | (100.0%) | 119 | (96.0%) |  |
| Yes | 5 | (5.2%) | 0 | (0.0%) | 5 | (4.0%) |  |
|  |  |  |  |  |  |  |  |
| Anthropometric characteristics |  |  |  |  |  |  |  |
| BMI (mean ± SD) | 31.5 | 5.2 | 31.5 | 5.3 | 31.5 | 5.2 | T-test    p= 0.68 |
| Abdominal circumference (mean ± SD) | 95.4 | 14.8 | 96.7 | 15.0 | 95.7 | 14.8 | T-test    p= 0.98 |

**Supplementary Table 3.** Effectiveness of the health-e-coaching intervention compared to standard care on BMI and abdominal circumference

|  | **Group** | | | |  | p |
| --- | --- | --- | --- | --- | --- | --- |
|  | **Standard care** | | **Health e-coaching** | | **Comparison*** |  |
|  | **N=51** | | **N=49** | | **β [95% CI]** |  |
| BMI (kg/m²) |  |  |  |  |  |  |
| T0 |  |  |  |  |  |  |
| N / Missing | 51 / 0 | | 49 / 0 | |  |  |
| Mean ± SD | 32.5 ± 5.2 | | 30.4 ± 5.1 | |  |  |
| Change: T1-T0 |  |  |  |  |  | **0.01** |
| N / Missing | 51 / 0 | | 49 / 0 | | 0 |  |
| Mean [95% CI] | **-0.9 [-1.5; -0.2]** | | 0.3 [-0.3; 1.0] | | **1.2 [0.3 ; 2.1]** |  |
| Abdominal circumference (cm) |  |  |  |  |  |  |
| T0 |  |  |  |  |  |  |
| N / Missing | 46 / 5 | | 46 / 3 | |  |  |
| Mean ± SD | 97.9 ± 16.7 [92.9; 102.8] | | 92.9 ± 12.4 [89.2; 96.6] | |  |  |
| Change: T1-T0 |  |  |  |  |  | 0.08 |
| N / Missing | 29 / 22 | | 29 / 20 | | 0 |  |
| Mean [95% CI] | -3.4 [-7.0; 0.2] | | 1.1 [-2.5; 4.7] | | 4.5 [-0.6; 9.6] |  |

* β=linear regression coefficient

**Supplementary Table 4.** Effectiveness of the health-e-coaching intervention compared to standard care on eating disorders

|  | **Group** | | | |  | **p** |
| --- | --- | --- | --- | --- | --- | --- |
|  | **Standard care** | | **Health e-coaching** | | **Comparison*** |  |
|  | **N=51** | | **N=49** | | **β [95% CI]** |  |
| Dieting (0-100) |  |  |  |  |  |  |
| T0 |  |  |  |  |  |  |
| N / Missing | 50 / 1 | | 49 / 0 | |  |  |
| Mean ± SD | 25.8 ± 17.8 | | 29.9 ± 19.7 | |  |  |
| Change: T1-T0 |  |  |  |  |  | 0.29 |
| N / Missing | 39 / 12 | | 41 / 8 | | 0 |  |
| Mean [95% CI] | 1.8 [-3.3; 7.0] | | -2.0 [-7.0; 3.0] | | -3.9 [-11.0; 3.3] |  |
| Bulimia (0-100) |  |  |  |  |  |  |
| T0 |  |  |  |  |  |  |
| N / Missing | 50 / 1 | | 49 / 0 | |  |  |
| Mean ± SD | 14.0 ± 16.4 | | 18.3 ± 19.7 | |  |  |
| Change: T1-T0 |  |  |  |  |  | 0.21 |
| N / Missing | 39 / 12 | | 41 / 8 | | 0 |  |
| Mean [95% CI] | 0.4 [-5.5; 6.3] | | -4.7 [-10.5; 1.0] | | -5.2 [-13.4; 3.0] |  |
| Oral control (0-100) |  |  |  |  |  |  |
| T0 |  |  |  |  |  |  |
| N / Missing | 50 / 1 | | 49 / 0 | |  |  |
| Mean ± SD | 3.9 ± 5.6 | | 3.9 ± 6.0 | |  |  |
| Change: T1-T0 |  |  |  |  |  | 0.77 |
| N / Missing | 39 / 12 | | 41 / 8 | | 0 |  |
| Mean [95% CI] | **2.9 [0.5; 5.3]** | | **3.4 [1.1; 5.8]** | | 0.5 [-2.8; 3.8] |  |
| Total score (0-100) |  |  |  |  |  |  |
| T0 |  |  |  |  |  |  |
| N / Missing | 50 / 1 | | 49 / 0 | |  |  |
| Mean ± SD | 17.2 ± 11.8 | | 20.2 ± 13.5 | |  |  |
| Change: T1-T0 |  |  |  |  |  | 0.25 |
| N / Missing | 39 / 12 | | 41 / 8 | | 0 |  |
| Mean [95% CI] | 1.8 [-1.8; 5.5] | | -1.2 [-4.7; 2.4] | | -3.0 [-8.1; 2.1] |  |

* β=linear regression coefficient

**Supplementary Table 5.** Effectiveness of the health-e-coaching intervention compared to standard care on anxiety and depression

|  | **Group** | | | |  | **Test** |
| --- | --- | --- | --- | --- | --- | --- |
|  | **Standard care** | | **Health e-coaching** | | **Comparison*** |  |
|  | **N=51** | | **N=49** | | **β [95% CI]** |  |
| Anxiety (0-100) |  |  |  |  |  |  |
| T0 |  |  |  |  |  |  |
| N / Missing | 49 / 2 | | 49 / 0 | |  |  |
| Mean ± SD | 40.1 ± 20.7 | | 43.7 ± 20.5 | |  |  |
| Change: T1-T0 |  |  |  |  |  | 0.74 |
| N / Missing | 35 / 16 | | 38 / 11 | | 0 |  |
| Mean [95% CI] | -2.9 [-9.3; 3.6] | | -1.4 [-7.6; 4.8] | | 1.5 [-7.5; 10.4] |  |
| Depression (0-100) |  |  |  |  |  |  |
| T0 |  |  |  |  |  |  |
| N / Missing | 46 / 5 | | 47 / 2 | |  |  |
| Mean ± SD | 23.9 ± 16.0 | | 26.6 ± 21.0 | |  |  |
| Change: T1-T0 |  |  |  |  |  | 0.94 |
| N / Missing | 34 / 17 | | 37 / 12 | | 0 |  |
| Mean [95% CI] | -2.7 [-9.4; 4.1] | | -3.0 [-9.4; 3.5] | | -0.3 [-9.7; 9.1] |  |
| Total score (0-100) |  |  |  |  |  |  |
| T0 |  |  |  |  |  |  |
| N / Missing | 46 / 5 | | 47 / 2 | |  |  |
| Mean ± SD | 32.5 ± 16.7 | | 34.8 ± 17.5 | |  |  |
| Change: T1-T0 |  |  |  |  |  | 0.70 |
| N / Missing | 33 / 18 | | 36 / 13 | | 0 |  |
| Mean [95% CI] | -3.2  [-9.0; 2.7] | | -1.6 [-7.2; 4.0] | | 1.6 [-6.5; 9.7] |  |

* β=linear regression coefficient

**Supplementary Table 6.** Effectiveness of the health-e-coaching intervention compared to standard care on quality of life

|  | **Group** | | | |  | **p** |
| --- | --- | --- | --- | --- | --- | --- |
|  | **Standard care** | | **Health e-coaching** | | **Comparison** |  |
|  | **N=51** | | **N=49** | | **β [95% CI]** |  |
|  |  |  |  |  |  |  |
| Physical Component Summary (0-100) | |  |  |  |  |  |
| T0 |  |  |  |  |  |  |
| N / Missing | 49 / 2 | | 49 / 0 | |  |  |
| Mean ± SD | 49.8 ± 9.0 | | 49.8 ± 10.1 | |  |  |
| Change: T1-T0 |  |  |  |  |  | 0.40 |
| N / Missing | 35 / 16 | | 39 / 10 | | 0 |  |
| Mean [95% CI] | 2.2 [-0.4; 4.8] | | 0.6 [-1.8; 3.1] | | -1.5 [-5.2; 2.1] |  |
| Mental Component Summary (0-100) | |  |  |  |  |  |
| T0 |  |  |  |  |  |  |
| N / Missing | 49 / 2 | | 49 / 0 | |  |  |
| Mean ± SD | 43.9 ± 8.5 | | 41.1 ± 6.9 | |  |  |
| Change: T1-T0 |  |  |  |  |  | 0.06 |
| N / Missing | 35 / 16 | | 39 / 10 | | 0 |  |
| Mean [95% CI] | -0.1 [-3.0 ; 2.7] | | **3.6 [0.9; 6.3]** | | 3.8 [-0.2; 7.7] |  |

* β=linear regression coefficient
